# The Long-Term Effects of Barren Land Afforestation on Plant Productivity, Soil Fertility, and Soil Moisture in China: A Meta-Analysis

**DOI:** 10.3390/plants13121614

**Published:** 2024-06-11

**Authors:** Yanqi Liu, Fucang Qin, Long Li, Xiaoyu Dong, Linfu Liu, Liangping Yang

**Affiliations:** 1 College of Desert Control Science and Engineering, Inner Mongolia Agricultural University, Hohhot 010018, China; yanqi930319@emails.imau.edu.cn (Y.L.); lilongdhr@imau.edu.cn (L.L.); dongxiaoyu2019@imau.edu.cn (X.D.); fuzlzz@emails.imau.edu.cn (L.L.); 2 Tongliao Forestry and Grassland Bureau Horqin District Branch, Tongliao 028000, China; 3 Forestry and Grassland Bureau of Inner Mongolia, Hohhot 010010, China; 4 Key Laboratory of National Forestry and Grassland Bureau for Desert Ecosystem Protection and Rehabilitation, Hohhot 010019, China; 5 Geological Survey Academy of Inner Mongolia Autonomous Region, Hohhot 010020, China; ylp225888@126.com

**Keywords:** barren land, afforestation, plant productivity, ecological restoration, soil fertility, soil moisture

## Abstract

As global ecological degradation intensifies, the long-term impacts of afforestation on productivity and soil fertility in barren lands have become critical in improving global ecological security and productivity. Through meta-analysis, this study integrates data from 109 barren land afforestation sites across China, aiming to comprehensively analyze the effects on plant productivity and soil fertility while identifying the key environmental drivers of these changes. We found that afforestation consistently enhances plant productivity across 60 years. However, soil fertility and moisture initially surged significantly after afforestation but gradually declined after the first decade, indicating the limited long-term benefits. Climatic factors, namely precipitation and humidity index, are crucial in enhancing plant productivity, while geographic factors, specifically lower elevations and gentler slopes, are associated with greater increases in soil fertility. Elevation and slope are two key factors that influence soil moisture after afforestation. These findings highlight the need for ongoing soil management and ecological maintenance in afforestation projects to sustain the soil fertility benefits. Our study provides a robust scientific foundation for afforestation strategies aimed at barren land restoration and offers valuable insights for policy formulation in barren land afforestation.

## 1. Introduction

Barren land, approximately 308 million hectares globally, accounted for 28% of the dry land that covers 41% of the Earth’s land surface [1]. Barren lands are predominantly located in Northern Africa and Central and Western Asia, regions that are primarily inhabited by impoverished populations [1]. Barren land is currently unused land, including hard-to-use land, i.e., degraded land, sandy land, bare land, mining land, etc., [2,3] exhibiting low productivity, poor soil fertility, and a drought condition. In the past decades, anthropogenic activities have led to the significant expansion of barren land, threatening global ecosystem productivity and safety [4,5]. Through restoration, the barren land could be transformed into farmland, grassland ecosystem, forest, etc., which is essential to global food and ecological security by providing more ecosystem services [6,7]. Afforestation, a widely recognized and implemented ecological restoration program, is paramount to revitalizing degraded ecosystems.

In China, the hyperarid zone spans about 50 million hectares, accounting for 6% of drylands [1]. China has implemented afforestation since the 1970s to restore degraded lands and has achieved great success. Approximately 46% of China’s drylands have been significantly improved or greened [8]. However, previous research efforts have predominantly focused on the effects of afforestation in agricultural lands or forest restoration domains [9,10]. The effects of afforestation on improving productivity, soil fertility, and soil moisture of barren lands have yet to be known.

The use of afforestation to enhance plant productivity and soil fertility in barren lands has attracted significant attention. Several studies have demonstrated that afforestation can significantly increase vegetation coverage and biomass [11,12]. Similarly, research by Dong et al. [13] has identified the positive impact of afforestation on soil fertility. However, these studies are constrained by limited observations in specific locations, lacking a comprehensive understanding of afforestation effects on a large scale and long-term benefits. Therefore, the conclusions of these studies inadequately reveal whether afforestation can effectively and continuously improve plant productivity and soil fertility. Understanding the effects of afforestation on barren land restoration at long-term scales is essential to restoration policymaking and practices [14,15].

Afforestation can lead to significant changes in soil moisture dynamics. Afforestation causes a decrease in soil water content, especially in deeper soil layers, due to the higher water uptake by tree roots and increased evapotranspiration compared to the original vegetation [16,17]. Research in Northeastern China showed that afforestation with species like *Pinus* and *Populus* spp. significantly reduced soil moisture content in the upper 100 cm of soil. The reduction varied across different depths, with the most pronounced decreases observed in deeper soil layers (30–100 cm) [17]. Studies in the Loess Plateau of China indicated that afforestation caused a strong decline in soil moisture below 2.2 m [18]. However, the long-term effects of barren land afforestation on soil moisture content in the topsoil at the national (China) scale have yet to be known.

Another crucial issue lies in the factors influencing the effects of barren land afforestation on plant productivity, soil fertility, and soil moisture. Previous studies have predominantly focused on the impacts of environmental control on trees planted on agricultural or grassland [19,20,21]. However, there has been limited research on environmental factors such as climate and topography and their relative influence on plant productivity, soil fertility, and soil moisture following afforestation in barren lands. For example, recent research indicates that topographical factors such as elevation and slope may significantly influence the benefits of afforestation. Specifically, higher elevation and steeper slopes may restrict nutrient cycling and soil conservation, thereby inhibiting improvements in soil fertility [20].

Additionally, climate factors may also play vital roles in the restoration process. For instance, adequate precipitation and suitable temperatures contribute to providing the necessary conditions for plant growth, exerting positive effects on vegetation coverage and productivity [19,22]. Therefore, a thorough investigation into the relative importance of these key factors on plant productivity and soil fertility long-term benefits is crucial for a comprehensive understanding of the restoration mechanisms of barren land ecosystems.

Understanding the effects of afforestation on barren lands is essential to global ecosystem health and sustainable development. Specifically, this study aims to identify the long-term effects and the influencing factors of barren land afforestation on plant productivity, soil fertility, and soil moisture through a national scale (China) meta-analysis. This study aims to comprehensively understand the feasibility and ecological benefits of afforestation on barren lands.

## 2. Results

### 2.1. Effects of Afforestation on Productivity and Soil Fertility in Barren Lands

**Figure 1:**
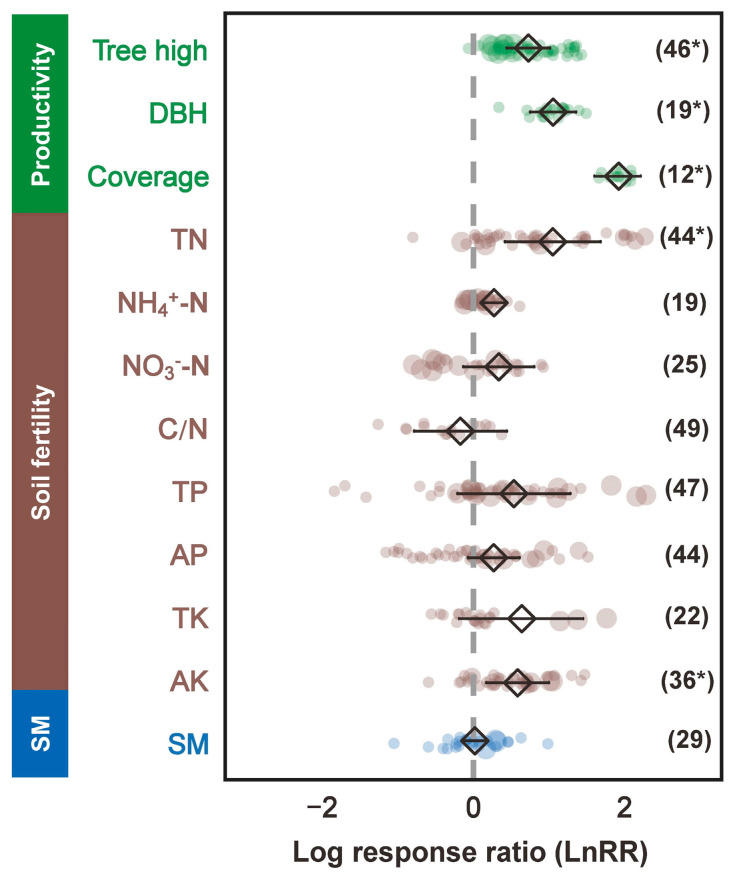
Responses of plant productivity, soil fertility, and soil moisture indicators to barren land afforestation. The vertical line was drawn at LnRR = 0. Error bars represent 95% bootstrap confidence intervals (CIs). A response was significant (marked with *) when the CI did not overlap with zero. DBH—Diameter at breast height; C/N—Carbon nitrogen ratio; TN—Total nitrogen; NH_4_^+^N—Ammonium nitrogen; NO_3_^−^N—Nitrate nitrogen; TP—Total phosphorus; AP—Available phosphorus; TK—Total potassium; AK—Available potassium; SM—soil moisture.

The results highlight the significant positive impact of tree height on productivity, emphasizing the importance of vertical growth in forest ecosystems. Afforestation significantly (*p* < 0.05) increased the tree height, diameter at breast height (DBH), and canopy coverage, suggesting a substantial supply capacity of afforestation activities for plant productivity (Figure 1). Soil fertility factors generally showed neutral to slightly positive effects, indicating their supportive but not overwhelmingly critical role in productivity. Specifically, concentrations of total nitrogen (TN), ammonium nitrogen (NH_4_^+^-N), and available potassium (AK) were significantly (*p* < 0.05) increased after afforestation, while other fertility-related indices also presented positive, albeit not significant, changes (Figure 1). Soil moisture demonstrated a non-significant (*p* > 0.05) effect, suggesting that higher soil moisture levels might have a marginally adverse impact on productivity, potentially due to waterlogging or reduced aeration (Figure 1).

### 2.2. Long-Term Effects of Afforestation on Productivity and Soil Fertility in Barren Lands

**Figure 2:**
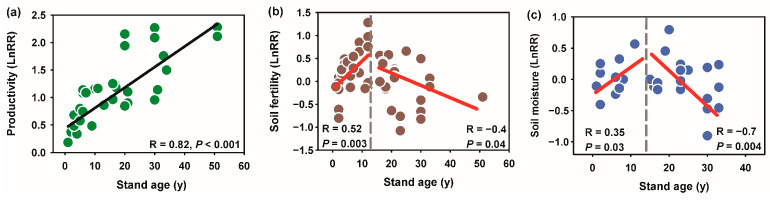
Nonlinear responses of plant productivity (**a**), soil fertility (**b**), and soil moisture (**c**) to stand age after barren land afforestation. Red solid lines represent the smoothed trend fitted at both sides of each threshold, respectively. Inset numbers in red and the vertical dashed lines describe the restoration age threshold identified.

The productivity of stands, measured as the LnRR (Formula (1)), exhibited a strong positive correlation with stand age (R = 0.82, *p* < 0.001) (Figure 2). The regression line indicates a consistent increase in productivity as the stand age increases, with a notable rise in productivity observed in stands up to approximately 50 years of age. This trend suggests that older stands tend to be more productive. Soil fertility, also measured as LnRR, showed a more complex relationship with stand age. A moderate negative correlation (R = −0.4, *p* = 0.04) existed between soil fertility and stand age beyond approximately one decade. Initially, soil fertility increased with stand age up to around 10 years (R = 0.52, *p* = 0.003), after which a decline was observed. This indicates that younger stands might benefit from increased soil fertility, but this benefit diminishes in older stands. The relationship between soil moisture and stand age was characterized by a biphasic trend, increasing before decreasing. For stands younger than one decade, there was a positive correlation between soil moisture and stand age (R = 0.35, *p* = 0.03). However, for stands older than one decade, a strong negative correlation was observed (R = −0.7, *p* = 0.004). This suggests that soil moisture tends to increase with a stand age of up to one decade, after which it significantly decreases. These findings highlight the dynamic interactions between stand age and key ecological factors, suggesting that forest management practices should consider the changing needs and conditions of stands as they age.

### 2.3. Environmental Drivers of Productivity and Soil Fertility Changes in Barren Land Afforestation

**Figure 3:**
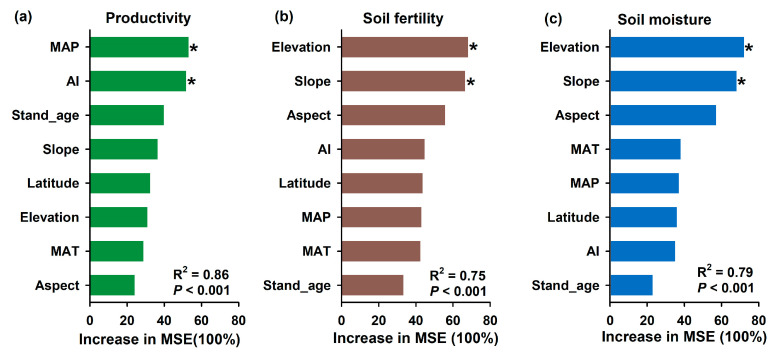
The random forest modeling identifies the relative influences (% of increase of mean square error (MSE)) of environmental factors on productivity (**a**), soil fertility (**b**), and soil moisture (**c**) following barren land afforestation. Significance level is * *p* < 0.05. MAP—Mean Annual Precipitation; MAT—Mean Annual Temperature; AI—Aridity index.

The influence of various environmental factors on productivity, soil fertility, and soil moisture following barren land afforestation was analyzed using random forest modeling. The relative influences of these factors are expressed as the percentage increase in mean square error (MSE). The models for productivity, soil fertility, and soil moisture exhibited high explanatory power with R^2^ values of 0.86, 0.75, and 0.79, respectively (*p* < 0.001) (Figure 3). The model explained 86% of the variance in productivity, where mean annual precipitation (MAP) had the highest impact on productivity, with a substantial increase in MSE. In addition, the aridity index (AI) also significantly influenced productivity, ranking just below MAP. The model explained 75% of the variance in soil fertility. Elevation had the highest impact on soil fertility, significantly increasing MSE. The slope was the second most influential factor, affecting soil fertility substantially. The model explained 79% of the variance in soil moisture. Elevation had the most substantial increase in MSE, indicating a significant impact on soil moisture. Slope also significantly influenced soil moisture. Random forest modeling identified MAP and AI as the most critical factors for productivity, with stand age, slope, and latitude also playing significant roles. For soil fertility, elevation and slope were the primary drivers, while aspect and AI had moderate influences. Soil moisture was predominantly affected by elevation and slope, followed by aspect and MAT. These findings underscore the importance of specific environmental factors in shaping ecological outcomes following barren land afforestation and provide guidance for optimizing forest management practices.

**Figure 4:**
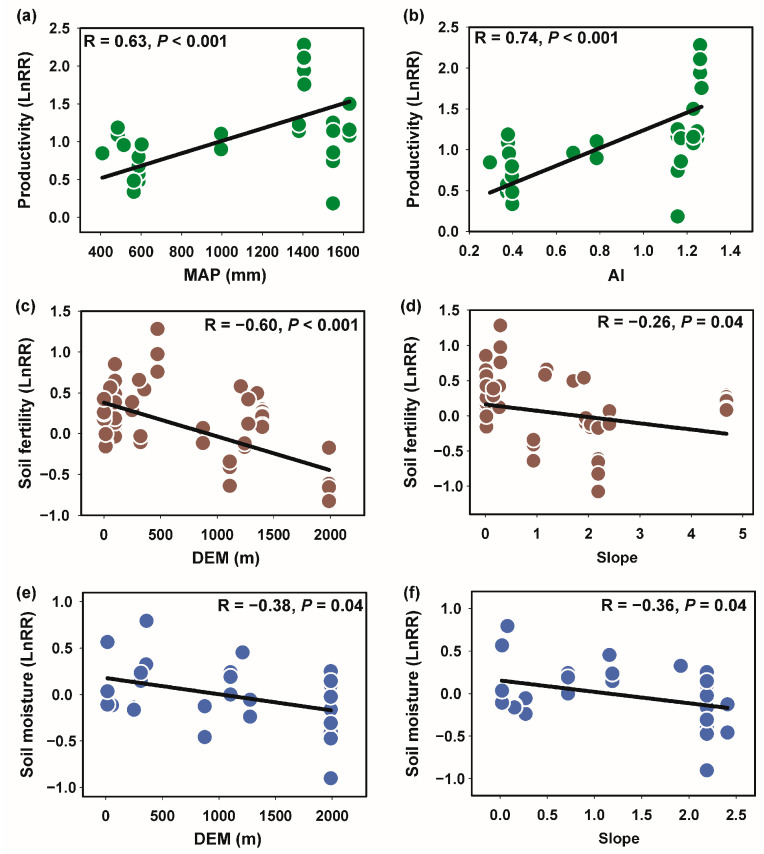
Key drivers associated with plant productivity, soil fertility, and soil moisture following barren land afforestation. Correlations between MAP (**a**)/AI (**b**) and productivity, between Elevation (**c**)/Slope (**d**) and soil fertility, and between Elevation (**e**)/Slope (**f**) and soil moisture. MAP—Mean Annual Precipitation; AI—Aridity index.

Further Pearson correlation analysis was applied to analyze the relationship between key environmental drivers and plant productivity, soil fertility, and soil moisture following barren land afforestation (Figure 4). There is a positive correlation between MAP and productivity (R = 0.63, *p* < 0.001) (Figure 4a). Higher levels of MAP are associated with increased productivity, indicating that precipitation is a crucial factor in enhancing plant growth. Figure 4b demonstrates a strong positive correlation between AI and productivity (R = 0.74, *p* < 0.001). Higher aridity indices are linked to greater productivity, suggesting that regions with moderate aridity benefit from increased plant growth following afforestation. A significant negative correlation was detected between elevation and soil fertility (R = −0.60, *p* < 0.001) (Figure 4c). Higher elevations are associated with reduced soil fertility, suggesting that altitude negatively affects nutrient availability or soil quality. Figure 4d shows a negative correlation between slope and soil fertility (R = −0.26, *p* = 0.04). Steeper slopes are linked to lower soil fertility, possibly due to increased erosion or reduced soil depth in sloped areas. Figure 4e shows a significant negative correlation between elevation and soil moisture (R = −0.38, *p* = 0.04). Higher elevations are associated with lower soil moisture, possibly due to increased drainage or lower precipitation retention at higher altitudes. Figure 4f indicates a negative correlation between slope and soil moisture (R = −0.36, *p* = 0.04). Steeper slopes correspond to reduced soil moisture, likely due to enhanced runoff and reduced water infiltration. The analysis reveals that MAP and AI are key positive drivers of productivity following barren land afforestation, while elevation and slope negatively impact soil fertility and soil moisture. These findings highlight the importance of considering these environmental factors in afforestation projects to optimize plant growth and soil health. The results underscore the need for targeted management practices that address specific environmental constraints to maximize the benefits of afforestation.

## 3. Discussion

### 3.1. Long Terms Effects of Barren Land Afforestation

Our study substantiates the substantial ecological benefits of afforestation on barren lands, particularly highlighting the long-term productivity gains. Such results suggest that tree species with strong adaptability more effectively utilize limited light and moisture resources, thereby accelerating growth in barren landscapes [23]. As vegetation proliferates, the expansion of leaf area in these trees enhances photosynthetic efficiency and carbon sequestration, which is crucial for biomass accumulation [24,25]. This not only boosts forest productivity but also improves microclimatic conditions at the ground level by expanding canopy cover, which helps retain soil moisture and reduces soil erosion [26]. These findings underscore the importance of afforestation strategies in barren areas of China for achieving sustained vegetation restoration and ecosystem functionality. Through these strategies, it is possible to restore and enhance productivity and increase the overall resilience and recovery capacity of ecosystems, marking a significant step towards sustainable management and ecological restoration of barren lands [27,28].

Afforestation on barren lands initially enhances soil fertility, reflecting the positive effects of vegetation restoration on soil quality. Early improvements in fertility primarily arise from the biological activities of newly planted trees, such as root development and leaf litter accumulation, which enrich soil organic matter and promote microbial activity [29]. This increases the soil content of key nutrients, including total nitrogen, ammonium nitrogen, and available potassium. However, particularly in inherently nutrient-poor and water-scarce barren environments, the original infertile state of the soil offers limited support for sustained vegetation growth in the long term [30]. Initial poor soil conditions, characterized by loose soil structure and low organic matter content, restrict the long-term nutrient supply capacity [30]. As vegetation matures, surface soil nutrients may gradually deplete, and it is unable to recover quickly through natural processes, especially in the absence of external inputs like fertilization [31].

Moreover, competition among mature vegetation roots intensifies, potentially increasing water and nutrient availability in deeper soil layers but reducing surface availability, further impacting topsoil fertility [32]. This phenomenon highlights that relying solely on initial vegetation recovery may not suffice for long-term soil fertility maintenance in barren land afforestation projects [33]. Therefore, comprehensive soil management practices are recommended to promote the long-term stability and sustainable development of barren land ecosystems. These include regular application of organic fertilizers, improvements in soil structure and water retention capabilities, and selection of plant species more adapted to arid conditions, all of which can help maintain and enhance soil fertility [33].

Interestingly, the changing trend of plant productivity doesn’t match that of soil fertility. Mature trees exhibit enhanced photosynthetic efficiency due to natural selection and genetic adaptations, enabling them to sustain high productivity even in nutrient-deficient conditions. As trees mature, their root systems develop significantly, potentially facilitating more efficient nutrient absorption from deeper soil layers or other challenging soil conditions. However, the soil samples were mainly taken from the topsoil. Furthermore, trees may have evolved to thrive in lower nutrient environments, prompting adjustments in their growth strategies or physiological mechanisms to optimize resource utilization.

The analysis of soil moisture (Figure 2c) reveals a nonlinear response to stand age following barren land afforestation. The trend shows an initial positive correlation (R = 0.35, *p* = 0.03) between stand age and soil moisture up to approximately a decade. Beyond this threshold, the relationship reverses, demonstrating a strong negative correlation (R = −0.7, *p* = 0.004), indicating that soil moisture decreases significantly in older stands. These results suggest that afforestation initially enhances soil moisture content in the surface layer, likely due to increased organic matter input and improved soil structure, which enhances water retention. This positive effect is most pronounced in the early stages of stand development, up to about 10 years. This increase can be attributed to several mechanisms, including improved soil structure, increased organic matter input, and enhanced water infiltration. For example, studies have shown that afforestation can lead to higher soil organic carbon (SOC) content, which improves soil aggregation and porosity, thereby enhancing the soil’s ability to retain water [34]. However, as the stands mature, the demand for water from the trees increases. Similar results indicated severe soil water depletion and significant soil desiccation occurred after 12 years of afforestation [35]. Mature trees with extensive root systems can significantly deplete soil moisture, especially during dry periods [16,36]. Additionally, the closed canopy in older stands can reduce rainfall infiltration and increase interception losses, leading to decreased soil moisture availability. This pattern is consistent with findings from various studies indicating that afforestation can improve soil moisture initially, it may also lead to soil moisture deficits in older stands due to higher evapotranspiration rates and canopy interception [37]. In páramo ecosystems, where high soil moisture is crucial for slowing decomposition and promoting high carbon storage, the reduction in water retention following afforestation may be the primary factor contributing to carbon loss [38].

### 3.2. Influencing Factors of the Long-Term Effects of Barren Land Afforestation

Our research further reveals that climatic factors, particularly precipitation and humidity, are significant drivers of productivity changes following afforestation on barren lands. This finding not only highlights the critical role of moisture in tree growth and ecosystem functionality but also underscores the importance of climatic conditions in the vegetation restoration process. Firstly, precipitation is a fundamental limiting factor for plant growth, especially in barren environments where water resources are inherently scarce [19,22]. Increased precipitation directly enhances soil moisture availability, which is crucial for the survival and growth of saplings. Enhanced moisture improves the water status of plants, reducing water stress and thereby increasing the efficiency and duration of photosynthesis, which in turn promotes biomass accumulation [32,39].

Additionally, sufficient moisture enhances plant nutrient uptake, as it facilitates the dissolution of nutrients in the soil and their absorption by plant roots [33]. Secondly, humidity, or the overall moisture condition of the environment, also significantly influences plant growth [40]. Higher humidity levels typically mean higher atmospheric and soil moisture, which helps plants cope with arid conditions by reducing moisture evaporation and improving water use efficiency. In the context of barren land afforestation, higher humidity creates a microclimate more conducive to plant growth, especially during the vulnerable sapling stage when plants are particularly sensitive to environmental conditions. In summary, climatic factors, especially precipitation and humidity, play a decisive role in barren land afforestation projects. They significantly drive productivity by directly providing essential moisture and creating favorable growth conditions.

Conversely, we discovered that it is not climatic but rather topographic factors, particularly elevation and slope, that significantly regulate soil fertility. Lower elevations and gentler slopes generally favor increased soil fertility due to their enhanced capacity to retain moisture [20]. In these areas, gentler slopes provide sufficient moisture to support microbial activity and nutrient cycling [41]. Additionally, these regions experience relatively low soil erosion, allowing organic matter and nutrients to accumulate rather than being washed away by rainfall, thereby enhancing fertility. In contrast, higher elevations and steeper slopes may adversely affect soil quality and fertility [20,42]. In these areas, intense soil erosion tends to remove the fertile topsoil and nutrients, limiting the nutrient resources available to plants. Simultaneously, steep slopes accelerate water runoff, reducing the availability of moisture in the soil, which further restricts microbial activity and the bioavailability of nutrients [41,42,43]. These factors collectively lead to lower soil fertility in regions with high elevations and steep slopes.

In the past decade, numerous studies have explored the effects of afforestation on soil water dynamics, shedding light on the complex interactions between vegetation, soil, and climate. Afforestation significantly influences soil water dynamics, a critical factor for ecosystem sustainability. Deforestation decreased the soil’s moisture content, but it varied among the tree species [17]. Soil water consumption is lower with diversified tree species for afforestation [44,45]. Mean annual precipitation, mean annual temperature and initial soil moisture jointly influence soil moisture across all land use types along the precipitation gradient following afforestation [46]. According to the current study, elevation and slope are the primary factors that influence soil moisture content following afforestation (Figure 3c). Elevation has the most substantial impact on soil moisture, indicating that elevation significantly affects soil water content. Higher elevations often experience lower temperatures and different precipitation patterns, which can influence soil moisture retention. Areas with gentle slopes and fine-textured soils generally exhibit better water retention compared to steep, coarse-textured soils, which are prone to rapid runoff and erosion. Influenced by litter decomposition and root turnover, soil organic matter further enhances soil’s water-holding capacity.

### 3.3. Limitation and Outlook

While our research has made significant progress in revealing the impacts of afforestation on barren lands in terms of productivity and soil fertility, it does have some limitations. Firstly, our study observed that afforestation in barren lands is predominantly monocultural, and we lack observational data to compare whether mixed-species planting would offer greater ecological benefits. Secondly, our model primarily focused on large-scale environmental factors, which might not have adequately considered the impacts of microenvironmental factors such as soil physical properties and soil microbiology. These aspects suggest areas for future research to enhance our understanding of the ecological success of afforestation projects.

Future research should explore the ecological benefits of mixed-species afforestation to address these limitations. Multispecies plantings are posited to enhance biodiversity, boost ecosystem resilience, and facilitate nutrient cycling. Long-term, controlled trials comparing monocultures and mixed-species configurations are essential to substantiate these benefits [47]. Moreover, a comprehensive evaluation of afforestation’s impacts on soil fertility necessitates a deeper analysis of the soil microenvironment. Future studies should integrate advanced soil science techniques, such as high-throughput sequencing and detailed ecosystem modeling, to discern the nuanced effects of afforestation strategies on soil health and productivity. Expanding research methodologies and integrating multidisciplinary approaches will sharpen our strategies for barren land restoration and propel innovation in ecological restoration practices.

## 4. Materials and Methods

### 4.1. Data Compilation

**Figure 5:**
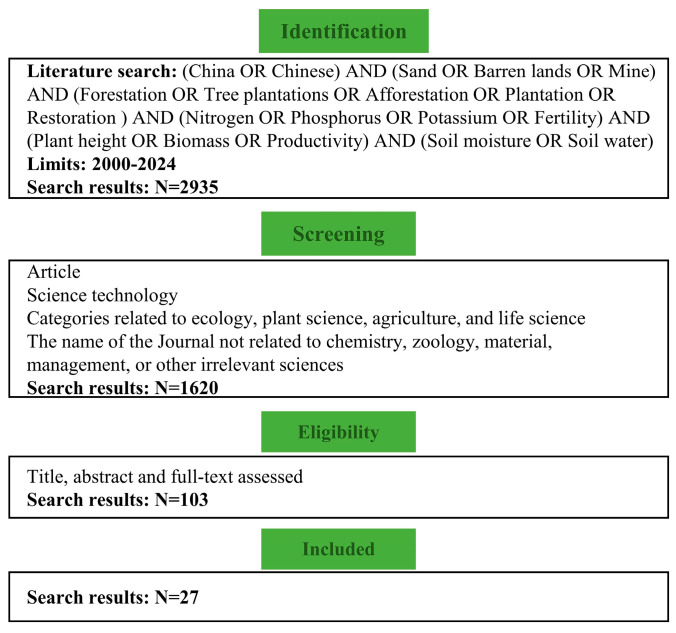
Article selection process using Preferred Reporting Items for Systematic Reviews (PRISMA) guidelines [48].

**Figure 6:**
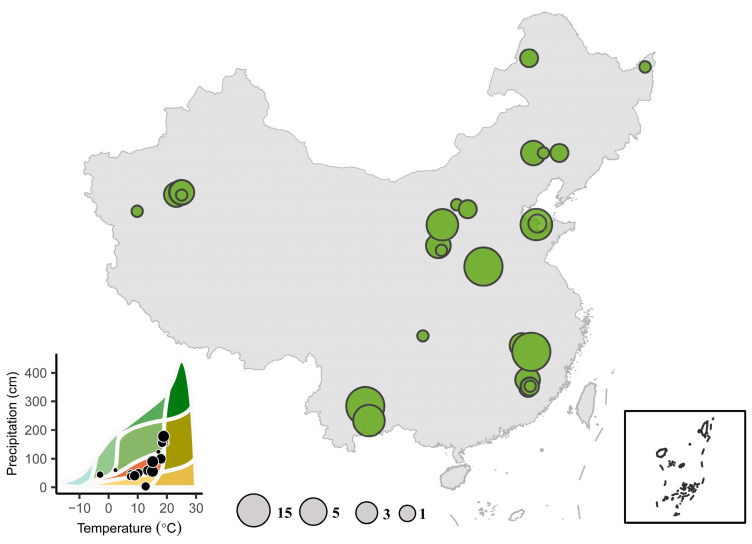
Geographical distribution of the study sites for barren land afforestation observations in China. Bubble size in maps is proportional to the amount of data for a given geographical location.

This study applied a meta-analysis method using journal articles from the Web of Science. We searched articles related to productivity and soil fertility under afforestation in barren lands with the following keyword combinations: “China” OR “Chinese” AND “sand” OR “bare land” OR “mine” OR “barren lands” AND “forestation” OR “tree plantations” OR “afforestation” OR “plantation” OR “restoration” AND “nitrogen” OR “phosphorus” OR “potassium” OR “fertility” AND “plant height” OR “biomass” OR “productivity” AND “soil moisture” OR “soil water”. The published dates of the articles are from January 2000 to April 2024. To avoid bias in publication selection, the following criteria are conducted to select the articles before being compiled in the database: (1) must provide at least one parameter of plant productivity or soil fertility; (2) the stand age of all stages should be clearly reported; (3) the mean values of selected variables in all stages can be extracted directly from table, digited graphs, or contexts. At last, 27 papers were selected to evaluate how afforestation influences productivity and soil fertility in barren lands of China (Figure 5). Articles that are used for meta-analysis are listed in Supplementary Materials. This study established a database of 109 barren land afforestation sites across China (Figure 6).

### 4.2. Indicators Used and Data Sources

Tree height, diameter at breast height, and canopy coverage were proxies for plant productivity. As for soil fertility, it was represented by the C/N (Carbon nitrogen ratio), TN (Total nitrogen), and NH_4_^+^-N (Ammonium nitrogen). NO_3_^−^N (Nitrate nitrogen), TP (Total phosphorus), AP (Available phosphorus), TK (Total potassium), and AK (Available potassium). The influencing factors include MAP (Mean Annual Precipitation), MAT (Mean Annual Temperature), Elevation, AI (Aridity index), stand age, slope, latitude, and aspect.

The MAP (Mean Annual Precipitation) and MAT (Mean Annual Temperature) were extracted from the WorldClim database (https://www.worldclim.org/, accessed on 3 April 2024) using the geographical coordinates of the study sites. For each site in the database, the AI (Aridity index) was calculated as the ratio of annual precipitation over potential evaporation (obtained from the WorldClim database). Elevation data was downloaded from ASTER Global DEM (https://asterweb.jpl.nasa.gov/GDEM.asp, accessed on 3 April 2024) using the geographical coordinates of the study sites. The aspect and slope for each site in the dataset were extracted using elevation. The data of the other parameters used in this study were directly extracted from the reviewed articles.

### 4.3. Statistical Analysis

#### 4.3.1. Response Ratio

The methods of Crouzeilles et al. [49] and Mooney et al. [50] were followed to calculate the *LnRR* of all ecosystem variables for all age combinations during forestation. The effect of afforestation on individual variables was estimated for each case study and calculated as the log response ratio (*LnRR*):

(1) $$LnRR=\ln\left(\frac{x_{t}}{x_{c}}\right)=\ln\left(x_{t}\right)-\ln\left(x_{c}\right)$$

where *x_t_* and *x*_c_ are the mean values of each elected variable in our study under forestation and reference ecosystem plots, respectively. The average *LnRR* values of soil fertility and productivity variables were calculated to represent the effects on soil fertility and productivity, respectively.

We used a linear mixed model in R package *me 4* with *LnRR* as the dependent variable to quantify the average effects of afforestation on plant productivity and soil fertility. The significance of these models was tested with likelihood ratio tests. Estimates of *LnRR* were derived from restricted maximum likelihood and 95% confidence intervals for the estimates obtained from the likelihood profile.

#### 4.3.2. Threshold Analysis

To evaluate the trend of plant productivity and soil fertility along stand ages. The presence of thresholds was explored when nonlinear regressions better fit the data. Two types of thresholds were explored, including a continuous threshold or a discontinuous threshold or breaking point, which refer to a continuous or abrupt change in each variable with stand age. We applied the *chngpt* and *segmented* packages in R to determine the thresholds [51]. Finally, we performed regression analysis on the data before and after the afforestation age threshold to determine the trend of plant productivity or soil fertility before and after the threshold. Here, R represents the Pearson correlation coefficient.

#### 4.3.3. Random Forest Analysis

Using random forest (RF) analysis, we identified the relative contribution of all fundamental factors to plant productivity or soil fertility: stand age, AI, MAT, MAP, elevation, slope, aspect, and elevation. The RF was implemented using the “*randomForest*” and “*importance*” functions of the *randomForest* package in R [52]. The mean squared error (MSE) represents the model’s prediction error, while R^2^ indicates the proportion of variance explained by the model.

Pearson analysis was conducted using the *cor.test* function in R to assess the relationships between significant predictors identified through random forest analysis and the parameters of plant productivity or soil fertility. Here, R represents the Pearson correlation coefficient. All data analyses were performed in R with geographic mapping facilitated by ArcGIS.

## 5. Conclusions

This study systematically examined the effects of afforestation on plant productivity, soil fertility, and soil moisture across barren lands in China. Analyzing data from 109 sites, we observed significant enhancements in tree diameter, height, and canopy coverage post-afforestation, particularly noteworthy increases in tree height, underscoring the efficacy of afforestation in bolstering productivity. However, while soil fertility improvements were substantial within the first decade post-afforestation, they gradually decreased thereafter, likely due to barren lands’ intrinsic poor soil quality and ongoing nutrient depletion. Soil moisture shares the same changing trend as soil fertility. Additionally, we identified precipitation and moisture levels as pivotal factors influencing plant productivity, whereas elevation and slope significantly affect soil fertility. Elevation and slope are key factors influencing soil moisture following afforestation. These findings stress the importance of accounting for geographic and climatic conditions in future afforestation efforts to optimize outcomes. Afforestation emerges as a potent ecological restoration tool, enhancing vegetative cover and ecosystem functionality in barren lands. Nevertheless, sustaining ecological benefits necessitates diligent post-afforestation soil management and nutrient supplementation. Our study provides a scientific framework for understanding the ecological restoration role of afforestation in barren lands and offers valuable guidance for shaping policy and implementing future afforestation initiatives.

## Data Availability

The original contributions presented in the study are included in the article/Supplementary Materials, further inquiries can be directed to the corresponding author/s.

## Supplementary Materials

The following articles are available online at https://www.mdpi.com/article/10.3390/plants13121614/s1, Supplementary File S1: Article used in the meta-analysis.

## References

[1] Patriarca C., Bako M., Branthomme A., Frescino T.S., Haddad F.F., Hamid A.H., Martucci A., Chour H.O., Patterson P.L., Picard N. (2019). Trees, Forests and Land Use in Drylands: The First Global Assessment.

[2] Guo Y., Abdalla M., Espenberg M., Hastings A., Hallett P., Smith P. (2021). A systematic analysis and review of the impacts of afforestation on soil quality indicators as modified by climate zone, forest type and age. Sci. Total Environ..

[3] Xu R., Wang Z., Zhu J. (2023). Land evolution in alpine inland river basin from 1980 to 2020 on the Northeastern Tibetan plateau, China. Appl. Ecol. Environ. Res..

[4] Bhagat S., Prasad P.R.C. (2024). Assessing the impact of spatio-temporal land use and land cover changes on land surface temperature, with a major emphasis on mining activities in the state of Chhattisgarh, India. Spat. Inf. Res..

[5] Wu S.Y., Liu L.B., Li D.L., Zhang W.T., Liu K.D., Shen J.S., Zhang L.B. (2023). Global desert expansion during the 21st century: Patterns, predictors and signals. Land Degrad. Dev..

[6] Qi K., Zhu J., Zheng X., Wang G.G., Li M. (2023). Impacts of the world’s largest afforestation program (Three-North Afforestation Program) on desertification control in sandy land of China. GIScience Remote Sens..

[7] Zipper C.E., Burger J.A., Skousen J.G., Angel P.N., Barton C.D., Davis V., Franklin J.A. (2011). Restoring Forests and Associated Ecosystem Services on Appalachian Coal Surface Mines. Environ. Manag..

[8] Li C., Fu B., Wang S., Stringer L.C., Wang Y., Li Z., Liu Y., Zhou W. (2021). Drivers and impacts of changes in China’s drylands. Nat. Rev. Earth Environ..

[9] Zhang G., Dong J., Xiao X., Hu Z., Sheldon S. (2012). Effectiveness of ecological restoration projects in Horqin Sandy Land, China based on SPOT-VGT NDVI data. Ecol. Eng..

[10] Ye L., Fang L., Shi Z., Deng L., Tan W. (2019). Spatio-temporal dynamics of soil moisture driven by ‘Grain for Green’ program on the Loess Plateau, China. Agric. Ecosyst. Environ..

[11] Han K.S., Park Y.Y., Yeom J.M. (2015). Detection of change in vegetation in the surrounding Desert areas of Northwest China and Mongolia with multi-temporal satellite images. Asia-Pac. J. Atmos. Sci..

[12] Wang X., Song J.L., Xiao Z.Q., Wang J., Hu F.Z. (2022). Desertification in the Mu Us Sandy Land in China: Response to climate change and human activity from 2000 to 2020. Geogr. Sustain..

[13] Dong L., Li J., Liu Y., Hai X., Li M., Wu J., Wang X., Shangguan Z., Zhou Z., Deng L. (2022). Forestation delivers significantly more effective results in soil C and N sequestrations than natural succession on badly degraded areas: Evidence from the Central Loess Plateau case. CATENA.

[14] Hayman G. (2024). Forestation is not an easy fix. Sciences.

[15] Bastin J.-F., Finegold Y., Garcia C., Mollicone D., Rezende M., Routh D., Zohner C.M., Crowther T.W. (2019). The global tree restoration potential. Science.

[16] Zhu X., Si J., He X., Jia B., Zhou D., Wang C., Qin J., Liu Z. (2024). Effects of long-term afforestation on soil water and carbon in the Alxa Plateau. Front. Plant Sci..

[17] Yao Y., Wang X., Zeng Z., Liu Y., Peng S., Zhu Z., Piao S. (2016). The effect of afforestation on soil moisture content in northeastern China. PLoS ONE.

[18] Zhang Q., Wei W., Chen L., Yang L. (2019). The joint effects of precipitation gradient and afforestation on soil moisture across the Loess Plateau of China. Forests.

[19] Wu X.T., Wang S., Fu B.J., Liu J.G. (2021). Spatial variation and influencing factors of the effectiveness of afforestation in China’s Loess Plateau. Sci. Total Environ..

[20] Guo J.H., Feng H.L., McNie P., Liu Q.Y., Xu X., Pan C., Yan K., Feng L., Goitom E.A., Yu Y.C. (2023). Species mixing improves soil properties and enzymatic activities in Chinese fir plantations: A meta-analysis. Catena.

[21] Berthrong S.T., Piñeiro G., Jobbagy E.G., Jackson R.B. (2012). Soil C and N changes with afforestation of grasslands across gradients of precipitation and plantation age. Ecol. Appl..

[22] Zhou G.Y., Zhou X.H., Eldridge D.J., Han X.M., Song Y.J., Liu R.Q., Zhou L.Y., He Y.H., Du Z.G., Delgado-Baquerizo M. (2022). Temperature and Rainfall Patterns Constrain the Multidimensional Rewilding of Global Forests. Adv. Sci..

[23] del Campo A.D., Segura-Orenga G., Ceacero C.J., González-Sanchis M., Molina A.J., Reyna S., Hermoso J. (2020). Reforesting drylands under novel climates with extreme drought filters: The importance of trait-based species selection. For. Ecol. Manag..

[24] Stephenson N.L., Das A.J., Condit R., Russo S.E., Baker P.J., Beckman N.G., Coomes D.A., Lines E.R., Morris W.K., Rüger N. (2014). Rate of tree carbon accumulation increases continuously with tree size. Nature.

[25] Avila R.T., de Almeida W.L., Costa L.C., Machado K.L.G., Barbosa M.L., de Souza R.P.B., Martino P.B., Juárez M.A.T., Marçal D.M.S., Martins S.C.V. (2020). Elevated air [CO2] improves photosynthetic performance and alters biomass accumulation and partitioning in drought-stressed coffee plants. Environ. Exp. Bot..

[26] Wang Q., Yao Y., Zhao L., Yang C.H., Zhao Y.C., Zhang Q.P. (2023). Enhancing resilience against geological hazards and soil erosion through sustainable vegetation management: A case study in Shaanxi Province. J. Clean. Prod..

[27] Li Y.R., Zhang X.C., Cao Z., Liu Z.J., Lu Z., Liu Y.S. (2021). Towards the progress of ecological restoration and economic development in China’s Loess Plateau and strategy for more sustainable development. Sci. Total Environ..

[28] Xu H., Yue C., Zhang Y., Liu D., Piao S.L. (2023). Forestation at the right time with the right species can generate persistent carbon benefits in China. Proc. Natl. Acad. Sci. USA.

[29] Giweta M. (2020). Role of litter production and its decomposition, and factors affecting the processes in a tropical forest ecosystem: A review. J. Ecol. Environ..

[30] Naorem A., Jayaraman S., Dang Y.P., Dalal R.C., Sinha N.K., Rao C.S., Patra A.K. (2023). Soil Constraints in an Arid Environment-Challenges, Prospects, and Implications. Agronomy.

[31] Stavi I., Islam K.R., Rahman M.A., Gusarov Y., Laham J., Comay O., Basson U., Xu C., Xu Z.W., Argaman E. (2023). Unexpected consequences of afforestation in degraded drylands: Divergent impacts on soil and vegetation. J. Environ. Manag..

[32] Cusack D.F., Addo-Danso S.D., Agee E.A., Andersen K.M., Arnaud M., Batterman S.A., Brearley F.Q., Ciochina M.I., Cordeiro A.L., Dallstream C. (2021). Tradeoffs and Synergies in Tropical Forest Root Traits and Dynamics for Nutrient and Water Acquisition: Field and Modeling Advances. Front. For. Glob. Change.

[33] Bowles T.M., Jackson L.E., Cavagnaro T.R. (2018). Mycorrhizal fungi enhance plant nutrient acquisition and modulate nitrogen loss with variable water regimes. Glob. Change Biol..

[34] Kahle P., Baum C., Boelcke B. (2005). Effect of afforestation on soil properties and mycorrhizal formation. Pedosphere.

[35] Liu Y., Miao H.T., Huang Z., Cui Z., He H.H., Zheng J.Y., Han F.P., Chang X.F., Wu G.L. (2018). Soil water depletion patterns of artificial forest species and ages on the Loess Plateau (China). For. Ecol. Manag..

[36] Li H., Li H., Wu Q., Si B., Jobbágy E.G., McDonnell J.J. (2023). Afforestation triggers water mining and a single pulse of water for carbon trade-off in deep soil. Agric. Ecosyst. Environ..

[37] Feng X.M., Fu B.J., Piao S., Wang S.H., Ciais P., Zeng Z.Z., Lü Y.H., Zeng Y., Li Y., Jiang X.H. (2016). Revegetation in China’s Loess Plateau is approaching sustainable water resource limits. Nat. Clim. Change.

[38] Farley K.A., Kelly E.F., Hofstede R.G.M. (2004). Soil organic carbon and water retention following conversion of grasslands to pine plantations in the Ecuadoran Andes. Ecosystems.

[39] Joshi J., Stocker B.D., Hofhansl F., Zhou S.X., Dieckmann U., Prentice I.C. (2022). Towards a unified theory of plant photosynthesis and hydraulics. Nat. Plants.

[40] Yu Z., Liu S., Li H., Liang J., Liu W., Piao S., Tian H., Zhou G., Lu C., You W. (2024). Maximizing carbon sequestration potential in Chinese forests through optimal management. Nat. Commun..

[41] Ma J.Y., Li Z.B., Ma B. (2020). Influences of revegetation mode on soil water dynamic in gully slope of the Chinese Loess hilly-gully region. Nat. Hazards.

[42] Al Omary A. (2011). Effects of aspect and slope position on growth and nutritional status of planted Aleppo pine (*Pinus halepensis* Mill.) in a degraded land semi-arid areas of Jordan. New For..

[43] Ma H., Zhu Q.K., Zhao W.J. (2020). Soil water response to precipitation in different micro-topographies on the semi-arid Loess Plateau, China. J. For. Res..

[44] Nan G.W., He X.Y., Ma L., Qin S.Y., Han L., Xu S.C. (2024). Identify a sustainable afforestation pattern for soil carbon sequestration: Considering both soil water-carbon conversion efficiency and their coupling relationship on the Loess Plateau. Land Degrad. Dev..

[45] Gong C., Tan Q.Y., Liu G.B., Xu M.X. (2024). Positive effects of mixed-species plantations on soil water storage across the Chinese Loess Plateau. For. Ecol. Manag..

[46] Liang H.B., Xue Y.Y., Li Z.S., Gao G.Y., Liu G.H. (2022). Afforestation may accelerate the depletion of deep soil moisture on the Loess Plateau: Evidence from a meta-analysis. Land Degrad. Dev..

[47] Feng Y.H., Schmid B., Loreau M., Forrester D., Fei S.L., Zhu J.X., Tang Z.Y., Zhu J.L., Hong P.B., Ji C.J. (2022). Multispecies forest plantations outyield monocultures across a broad range of conditions. Science.

[48] Moher D., Liberati A., Tetzlaff J., Altman D.G., Prisma Group (2009). Preferred reporting items for systematic reviews and meta-analyses: The PRISMA statement. Ann. Intern. Med..

[49] Crouzeilles R., Ferreira M.S., Chazdon R.L., Lindenmayer D.B., Sansevero J.B.B., Monteiro L., Iribarrem A., Latawiec A.E., Strassburg B.B.N. (2017). Ecological restoration success is higher for natural regeneration than for active restoration in tropical forests. Sci. Adv..

[50] Mooney K.A., Gruner D.S., Barber N.A., Van Bael S.A., Philpott S.M., Greenberg R. (2010). Interactions among predators and the cascading effects of vertebrate insectivores on arthropod communities and plants. Proc. Natl. Acad. Sci. USA.

[51] Fong Y.Y., Huang Y., Gilbert P.B., Permar S.R. (2017). chngpt: Threshold regression model estimation and inference. BMC Bioinform..

[52] Breiman L. (2001). Random forests. Mach. Learn..

