# Supplementary material for: The Long-Term Effects of Barren Land Afforestation on Plant Productivity, Soil Fertility, and Soil Moisture in China: A Meta-Analysis
Source: Plants (Basel). 2024 Jun 11;13(12):1614. doi: 10.3390/plants13121614 (PMC11207343; doi:10.3390/plants13121614)
Supplement: Supplementary file 1 [file plants-13-01614-s001.zip › plants-3041943-supplementary.pdf]

Supplementary Materials

Articles included in the meta-analysis

1. Yu, H.-X.; Wang, C.-Y.; Chen, H.; Tang, M., Dynamics of Ectomycorrhizal Fungal Communities in a Chronosequence of Pinus tabulaeformis Plantations. Journal of Pure and Applied Microbiology 2014, 8, (2), 1197-1208.

2. Zhang, Y.; Li, Y.; Wang, S.; Umbreen, S.; Zhou, C., Soil phosphorus fractionation and its association with soil phosphate-solubilizing bacteria in a chronosequence of vegetation restoration. Ecological Engineering 2021, 164, 106208.

3. Bowles, T. M.; Jackson, L. E.; Cavagnaro, T. R., Mycorrhizal fungi enhance plant nutrient acquisition and modulate nitrogen loss with variable water regimes. Global Change Biology 2018, 24, (1), E171-E182.

4. Li, J.; Awasthi, M. K.; Zhu, Q.; Chen, X.; Wu, F.; Wu, F.; Tong, X., Modified soil physicochemical properties promoted sequestration of organic and inorganic carbon synergistically during revegetation in desertified land. Journal of Environmental Chemical Engineering 2021, 9, (6), 106331.

5. Zhao, P. s.; Guo, M. s.; Gao, G. l.; Zhang, Y.; Ding, G. d.; Ren, Y.; Akhtar, M., Community structure and functional group of root-associated Fungi of Pinus sylvestris var. mongolica across stand ages in the Mu Us Desert. Ecology and Evolution 2020, 10, (6), 3032-3042.

6. Guo, M. s.; Ding, G. d.; Gao, G. l.; Zhang, Y.; Cao, H. y.; Ren, Y., Community composition of ectomycorrhizal fungi associated with Pinus sylvestris var. mongolica plantations of various ages in the Horqin Sandy Land. Ecological Indicators 2020, 110, 105860.

7. Cui, Y.; Yong, L.; Mo, R.; Wen, D.; Zhu, Z.; Hu, X., Metagenomics analysis of the effects of long-term stand age on beneficial soil bacterial community structure under Chinese ancient mulberry farming practice. Horticulture Environment and Biotechnology 2020, 61, (6), 1063-1071.

8. Sui, X.; Zhang, R.; Frey, B.; Yang, L.; Li, M.-H.; Ni, H., Land use change effects on diversity of soil bacterial, Acidobacterial and fungal communities in wetlands of the Sanjiang Plain, northeastern China. Scientific Reports 2019, 9, 18535.

9. Pang, D.; Cui, M.; Liu, Y.; Wang, G.; Cao, J.; Wang, X.; Dan, X.; Zhou, J., Responses of soil labile organic carbon fractions and stocks to different vegetation restoration strategies in degraded karst ecosystems of southwest China. Ecological Engineering 2019, 138, 391-402.

10. Li, Y.; Wang, X.; Wang, Z.; Du, Z.; Wang, S.; Liu, D., Soil microorganism and soil respiration characteristics of Tamarix chinensis plantations of different ages in the Yellow River Delta during the growing season. Journal of Central South University of Forestry & Technology 2019, 39, (2), 86-92.

11. Li, P.; Zhang, X.; Hao, M.; Cui, Y.; Zhang, Y.; Zhu, S., Effects of Vegetation Restoration on Soil Physicochemical Properties, Enzyme Activities,and Fungal Community of Reconstructed Soil in a Mining Area on Loess Plateau. Bulletin of Soil and Water Conservation 2019, 39, (5), 1-7.

12. Zhang, H.; Lu, M.; Xie, J., Effect of Dicranopteris dichotoma on soil microbial community structure in red soil erosion area. Acta Ecologica Sinica 2018, 38, (5), 1639-1649.

13. Ren, Q.; Li, C.; Yang, W.; Song, H.; Ma, P.; Wang, C.; Schneider, R. L.; Morreale, S. J., Revegetation of the riparian zone of the Three Gorges Dam Reservoir leads to increased soil bacterial diversity. Environmental Science and Pollution Research 2018, 25, (24), 23748-23763.

14. Li, Y.; Wang, Z.; Li, Z.; Chen, M.; Liu, D., Soil nutrients and biological characteristics of Tamarix chinensis plantations with different ages in the Yellow River Delta. Journal of Arid Land Resources and Environment 2018, 32, (4), 89-94.

15. Tian, Q.; Niu, C.; Taniguchi, T.; Yamanaka, N.; Shi, W.; Du, S., Relationship among vegetation types and soil microbial biomass in the Loess Hilly region of China. Acta Ecologica Sinica 2017, 37, (20), 6847-6854.

16. Pu, J.; Qi, Y.; Wang, Y.; Chu, W.; Yang, F., Effects of different plant communities on soil microbial biomass carbon, nitrogen and phosphorus in the agro-pastoral transitional zone of northern Shaanxi Province. Agricultural Research in the Arid Areas 2015, 33, (4), 279-285.

17. Li, J.; Zhou, X.; Yan, J.; Li, H.; He, J., Effects of regenerating vegetation on soil enzyme activity and microbial structure in reclaimed soils on a surface coal mine site. Applied Soil Ecology 2015, 87, 56-62.

18. Li, C.; Shi, L.-L.; Ostermann, A.; Xu, J.; Li, Y.; Mortimer, P. E., Indigenous trees restore soil microbial biomass at faster rates than exotic species. Plant and Soil 2015, 396, (1-2), 151-161.

19. Wang, S.; Zhao, X.; Zhang, T.; Li, Y.; Lian, J.; Huang, W.; Yun, J., Afforestation effects on soil microbial abundance, microbial biomass carbon and enzyme activity in dunes of Horqin Sandy Land, northeastern China. Sciences in Cold and Arid Regions 2013, 5, (2), 184-190.

20. Deng, H.; Zhang, B.; Yin, R.; Wang, H.-l.; Mitchell, S. M.; Griffiths, B. S.; Daniell, T. J., Long-term effect of re-vegetation on the microbial community of a severely eroded soil in sub-tropical China. Plant and Soil 2010, 328, (1-2), 447-458.

21. Zhong, J. I. N. Z.; Qiang, L. E. I. J.; Wen, X. U. X.; Yu, L. I. S.; Feng, Z. S., Microbial diversities of shelter-forest soils in the extreme arid area. Acta Ecologica Sinica 2009, 29, (8), 4548-4559.

22. Du, X.; Tang, M.; Chen, H.; Zhang, H.; Zhang, Y. a., Mycorrhizae and Diversity of Microbial Community in Rhizosphere Soils of Robinia pseudoacacia at Different Ages on the Loess Plateau. Scientia Silvae Sinicae 2008, 44, (4), 78-82.

23. Chengyou, C. A. O.; Lihui, Z. H. U.; Deming, J.; Yao, F. U.; Feifei, G. A. O., Effects of Artificial Sand-Fixation Communities on Soil Nutrients and Biological Properties in Horqin Sandy Land. Journal of Soil and Water Conservation 2007, 21, (1), 168-171.

24. Jin, Z. Z.; Lei, J. Q.; Li, S. Y.; Xu, X. W., Variation characteristics of soil microbial activities in the Tarim Desert Highway shelter forests, Xinjiang of Northwast China. The Journal of Applied Ecology 2013, 24, (9), 2464-2470.

25. Hou, X.; Han, H.; Tigabu, M.; Cai, L.; Meng, F.; Liu, A.; Ma, X., Changes in soil physico-chemical properties following vegetation restoration mediate bacterial community composition and diversity in Changting, China. Ecological Engineering 2019, 138, 171-179.

26. Du, Y.; Gao, G.; Chen, L.; Ding, G.; Zhang, Y.; Cao, H., Soil bacteria community structure and function prediction in the Hulun Buir Sandy Area. China Environmental Science 2019, 39, (11), 4840-4848.

27. Zhang, X. Y.; Xiong, S. Y.; Wu, X. K.; Zeng, B. B.; Mo, Y. M.; Deng, Z. C.; Wei, Q.; Gao, Y.; Cui, L. C.; Liu, J. P.; Long, H. Z., Dynamics of Microbial Community Structure, Function and Assembly Mechanism with Increasing Stand Age of Slash Pine (Pinus elliottii) Plantations in Houtian Sandy Area, South China. Journal of Microbiology 2023, 61, (11), 953-966.
